# Our centenary issue

**Published:** 2018-02-08

**The *Community Eye Health Journal* was created in 1988 to meet the needs of eye care workers worldwide. Thirty years and 100 issues later, our readers remain at the centre of all we do.**

**Figure:**
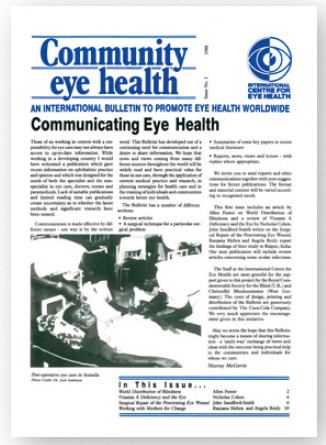


Blindness, visual impairment and eye disease affect people everywhere. However, not everyone is equally at risk; even in wealthier countries it is always the poor who suffer the most.

The history of the *Community Eye Health Journal* is deeply entwined with that of global eye health. We are the only publication specifically for eye care workers in low- and middle-income countries, and we have been here since 1988.

At this significant milestone, it seems appropriate to pause and reflect on the global story of eye health. In the pages that follow, our contributors examine what has been achieved over the last 30 years and the many challenges that still lie ahead.

But first I would like to put the spotlight on you: our readers. Your daily work is an essential part of the global effort to reduce avoidable blindness and visual impairment and to care for those whose vision cannot be improved. Everything you do is helping to end the needless suffering of those who cannot see well, who are in pain, or who feel that no-one cares.

And you are not alone. Thousands, if not millions, of people worldwide are also helping to improve the eye health of others. They may be treating patients, conducting research, maintaining equipment, balancing the books, working for the World Health Organization, fundraising or scrubbing the operating theatre. Everyone is part of this team.

It is a privilege to share our 100th issue with all of you. Thank you for everything you are doing, and long may you continue!

*Elmien Wolvaardt Ellison (Editor)*

